# Research in place: the critical role of rural health research in Canada

**DOI:** 10.3389/fmed.2025.1600660

**Published:** 2025-08-26

**Authors:** Stephanie Welton, Margo Wilson, Barbara Zelek, David Pontin, Grace Perez, Nelly D. Oelke, Aaron Johnston, Jill Konkin

**Affiliations:** ^1^Faculty of Medicine, Department of Family Medicine (South West Nova Site), Dalhousie University, Yarmouth, NS, Canada; ^2^Faculty of Medicine, Discipline of Emergency Medicine, Memorial University, St. John’s, NL, Canada; ^3^Section of Family Medicine, Dr. Gilles Arcand Centre for Health Equity, Northern Ontario School of Medicine University, Thunder Bay, ON, Canada; ^4^Faculty of Medicine and Dentistry, Department of Family Medicine (Yellowknife Site), University of Alberta, Yellowknife, NT, Canada; ^5^Cumming School of Medicine, Distributed Learning and Rural Initiatives, University of Calgary, Calgary, AB, Canada; ^6^School of Nursing, University of British Columbia Okanagan Campus, Kelowna, BC, Canada; ^7^Rural Coordination Centre of British Columbia, Vancouver, BC, Canada; ^8^Cumming School of Medicine, Distributed Learning and Rural Initiatives, Departments of Emergency Medicine and Family Medicine, University of Calgary, Calgary, AB, Canada; ^9^Faculty of Medicine and Dentistry, Department of Family Medicine, University of Alberta, Edmonton, AB, Canada

**Keywords:** research capacity building, rural health research, primary care research, rural medical education, distributed medical education

## Abstract

People in rural communities often experience different access to healthcare and services, which can lead to poorer health outcomes compared to their urban counterparts. This holds true across the international context, though our focus here is on Canada. Health research plays a crucial role in identifying challenges and solutions, and we argue that research conducted in rural communities by rural researchers is essential to addressing the unique needs of a rural population. However, several barriers hinder rural research in Canada, including inadequate infrastructure, uneven resource distribution, and the absence of a national rural research network. Prioritizing rural research is vital, as it can improve workforce recruitment and retention while guiding informed healthcare decisions and policies.

## Introduction

All Canadians have the right to universal and accessible health care as outlined in the Canada Health Act. Despite this, people living in rural and remote communities often face challenges accessing care in their home community (1, 2), and have worse health outcomes than patients in urban areas (3). Urban-based health research does not often translate to improved rural outcomes (4). The development of distributed medical education programs in Canada reflects a shift from urban academic centers to education in communities (5). While this distribution provides medical education in rural contexts, equivalent academic structures for rural-specific research are lacking. Rural health research is crucial to providing direction for health policy, and interventions for optimal patient outcomes (5–7).

Our objective for this paper is to support the vital role of rural health research, explore challenges in generating authentic rural evidence in Canada, and discuss barriers and assets toward building a robust national field of rural health research. We aim to address this by (1) examining successful rural health research strategies; (2) identifying barriers to growth in three key areas: infrastructure, workforce capacity, and connectivity, while illustrating approaches to overcome them; (3) describing a role for distributed health professions education to grow rural health research; and (4) proposing a shift in perspective to raise the profile of rural health research in Canada.

## Health research in the rural context

Rural communities are not uniform, and there are many ways to define rurality, each with pitfalls (8–11). Here, we will use the term “rural” to encompass rural, regional, remote, and northern settings (12). Each can be under-represented in the evidence base, due to historic under investment in research outside of urban academic settings (4, 13). Solutions facilitating community-based research in the smallest, most remote, most northern places can serve all rural populations and non-traditional research settings. Research conducted in rural Indigenous communities should meaningfully include community members throughout the process (14).

Leaders in rural research capacity building efforts in Canada point out that innovation is central to rural medicine (15). Rural physicians are driven to discover solutions to problems facing their communities (15). Conducting research in rural communities can be quite different from the approach of the urban academic setting. Rural health researchers in Australia work with many methodologies and topics, in small multidisciplinary teams, and trusting local partnerships. They build a generalist skillset to best serve community needs, and find their work highly rewarding because of its impact on their communities and health systems (16). This approach centers on social accountability. Research in rural settings must be done by rural people to generate meaningful evidence (17–19). Differences in rural demographics, geography, and service availability make urban evidence difficult to generalize (20–22).

Canadian rural physicians are scholars, interested and well positioned to carry out local research (13, 15). Their impact can be amplified by involvement of all health professions and local academically trained researchers (23–25). It can be difficult to conduct research in the rural context due to many competing priorities, in addition to geographic and professional isolation, which can lead to delays or abandonment of research work (26). System-wide supports are needed to facilitate research in this context: infrastructure, skill building, connection and integration.

## Rural research infrastructure

The infrastructure that regularly supports research in academic institutions is lacking in rural areas. A system-wide evaluation of rural research needs in one Australian jurisdiction describes an over-reliance on individual activities, resulting in fragmentation, and identifies a need for support at the systems level (27). Early career rural researchers from several communities describe having limited support or staff, and being responsible for all aspects of research processes, including finding opportunities to build capacity toward meeting high community demand (16). This is compounded by challenges typical of rural contexts (geography, demographics, service availability) and rural research (wide breadth of topics and methodologies) (16, 22). Fixed-term funding models for rural academics present a barrier to continuity of high-quality rural research and urban–rural partnership (28). In Canada, rural academia is not as well defined or established, though it faces similar obstacles (13, 15).

Rural health professionals infrequently have access to academic appointments, protected academic time, grant administrative support, research assistants, methodological support, or highly qualified research personnel. Instead, some universities have formed dedicated units for rural health research. In Canada, the Center for Rural Health Research at the University of British Columbia emerged due to demand for evidence to inform policy on health services delivery to serve rural communities (29). The Center for Rural Health Studies at Memorial University of Newfoundland performs similar work, with a particular focus on rural/urban disparities (30). These centers benefit from the research infrastructure of central universities, and generate important rural evidence.

Still, place-based research is a unique, under-explored concept. One success story is Colac, a rural town in Australia. There, local research activities generate evidence that influences practice, and this promotes growth in research interest, capacity, and funding. This successful research program began with one team project, leveraging temporary availability of PhD expertise to gain funding for research support and protected research time for clinicians. Program expansion has secured sustainable funding for support staff, and sees newly engaged clinician researchers bring in project grants (23). They advocate for expanding opportunities for research at similar sites, and exploring small rural hospitals as drivers of rural research (23, 24). Similarly, teams led from Sweden and America describe the central roles of “local-actors and local-action” and “local relevance” in rural health innovation (18, 19).

Rural physicians in Canada are also well positioned to lead research in their communities (15). In Canada, the distributed medical education networks of all medical schools present excellent scaffolding for rural health research that remains largely unexplored (13). Health professions education programs in Australia can access funding for dedicated rural research professionals at distributed sites. This has contributed substantially to building rural research capacity and establishing rural research networks, leading to improved service delivery, patient care, educational innovations, and workforce retention (28).

Yet, investment in rural health research does not reflect the demand for rural evidence. In Canada, 17% of the population lives rurally, while the Canadian Institutes for Health Research (CIHR) awarded less than 1% of funds to rural projects from 2000 to 2019 (4, 31). Rural-specific funding avenues have long been advocated for at the national level (4, 32). In 2007, the Canadian Rural Health Research Society named the dismantling of the Health Canada Rural Health Office and removal of rural funding priorities by the Canadian Institute of Health Research as key barriers (33). Rural organizations now provide funding for rural work, such as Rural360 for research trainees in rural Newfoundland, and the Rural Physician Research Grant Program in British Columbia (34, 35). These efforts cannot meet the demand for rural evidence alone. Yet the profile of a successful rural researcher includes key differences from their urban counterparts that at present have no mechanism to be recognized or awarded in major grant competitions, and may even put them at a disadvantage (16).

Rural health research in Canada has been driven by the enthusiasm of dedicated individuals, often working outside of their payment structure, or on contractual funding. It is time for the administrative and methodological supports to be based in rural communities, with protected academic time at distributed sites to facilitate and support collaborative multidisciplinary research teams, with access to funding that reflects the demand for rural evidence.

## Skill building to grow rural health research capacity

At present, the rural workforce does not have enough health professionals to meet clinical needs, and even fewer with the knowledge, expertise and skills to meet the demand for research (23). Workforce turnover is another barrier, and affects all health professions in rural communities. However, academic opportunities can support rural health workforce retention (6, 28, 36). Rural skill building programs in research are needed (37). To be truly meaningful, training must be done by rural people for rural people (12, 26). In Australia, despite having a more established role for rural academia, needs assessments call for training programs to build rural health research capacity, and develop more pathways to rural academia (12, 16, 27). Working rural academics also seek out skill building opportunities to develop a more generalist skill set to better meet the needs of their communities (16).

Memorial University of Newfoundland Canada has created a faculty development program to specifically build rural research capacity in medicine (26, 38). The program was designed to equip rural physicians to carry out research in their communities (37). Alumni are supported to continue their work with ongoing mentorship, research assistant support, and incorporation into a growing network of rural health researchers. The program’s impacts in capacity building extend beyond the new research skills and outputs of trainees, to also raising the profile of research in rural medicine across the participating region, and highlighting resources available to all faculty (26). Still, the output of this program is small in numbers in comparison to the need, and not enough similar initiatives are in place nationally in Canada. The program development team identifies Faculty of Medicine support and funds as a key facilitator for program initiation, along with a professional culture valuing homegrown rural evidence, leading to quick uptake (26).

University rural health research centers present opportunities for a variety of trainees, clinical and non-clinical, to conduct research with a rural focus. However, this will not necessarily equip trainees for place-based research. In-community research lags behind academic centers in learning opportunities. As demonstrated in Colac Australia, clinicians can be engaged in research through local projects, and thereby gain the skills to lead future work (23). Undergraduate and postgraduate medical trainees participate in scholarly work, and their learning opportunities in place-based research would also be enhanced by increased availability of local research expertise at rural teaching sites. Engaging with students on scholarly activities is another way for rural faculty to conduct and support research, and to foster the creation of scholars within the rural community (39).

Becoming a rural health researcher, in Australia, usually involves either a rural move for a specific role, or pursuing a PhD to get one, though a few move rurally for personal reasons and later find work in rural academia (16). They describe a need for additional pathways to rural academia to meet the high demand for rural health research (16). Canada remains without formal pathways to rural academia, and often depends on individual drive to bring research skills to communities. A systematic approach encouraging more health researchers in rural spaces is essential, involving specific rural health research training opportunities (16, 37), rural research trainee scholarships (16), and visibility of rural research projects (16, 26).

## National strategies to support growth in rural health research

In 2017, a joint task force of the College of Family Physicians of Canada and the Society of Rural Physicians of Canada created The Rural Roadmap for Action, aimed at improving the health of rural Canadians. Direction 4 calls for a national rural research agenda, rural health services research network, and strengthening rural medical education by incorporating research (40). While the Society of Rural Physicians of Canada is engaged in development efforts, success will require support from outside the rural space, and more can be done to include other health professions in rural research (1, 25, 41).

### Rural health research agenda

A national rural research strategy is needed that includes priority research topics and funding to support a national rural health research network and research activities. This can ensure a collective focus on relevant research and ensure it is shared with others, learning together from both the process and the results, and integrating rural evidence into an overall picture. Developing a national rural health research strategy will need to incorporate perspectives and priorities of rural health professionals, rural medical and health professions education programs, rural-based academics, and rural populations, as well as meaningful engagement and collaboration with Indigenous groups. Recently, the National Summit on Equitable Access to Medical Transport in Rural Canada identified interested parties and brought them together to understand priorities (42). Led by the Society of Rural Physicians of Canada, this approach outlines a strategy that could also be applied to shaping a health research agenda and strategy for rural Canada. Involvement from government and major funders would allow more sustainable solutions (4, 33). The United States provides a small-scale example, with federally funded short-term Rural Health Research Centers studying priority rural healthcare policy issues, though small in number and geographic coverage (43).

### Rural health research network

Canada is without a true rural research network, nationally. Rural health researchers are often working in relative isolation from their colleagues in research, and disconnected from other findings in their field (37). A national rural health research network can create connections, collaborations, visibility, and legitimacy. The Hauora Taiwhenua Rural Health Network has done this for New Zealand, by creating a research and education chapter, with a dedicated national gathering, and intentions to define national rural health research and education priorities (44). Several Canadian jurisdictions have mechanisms for sharing rural health research resources and information (45–47). These initiatives have risen to address local or regional needs but are unconnected to each other. A national network would strengthen existing rural health research organizations and better serve more geographically isolated communities.

### Rural research in distributed health professions education

Robust distributed medical education provides an ideal ground to grow rural research, as is already being done in Australia. Canadian medical schools have strong distributed networks, well positioned to grow rural research (5, 13). Strong medical education enhances research, and research enhances medical education (15). The NorFam residency program, based in Happy Valley-Goose Bay Labrador, is one of the earliest examples of rural and remote residency program delivery (48). This community is also home to the School of Arctic and Subarctic Studies, an important player in bringing socially accountable research to Canada’s North (49). Together, these parallel entities could open more opportunities for the population than either would alone, in establishing better access to care and creating an avenue for representative evidence and policy advocacy. Health professions’ education could intentionally create more such opportunities for the many communities involved in distributed education networks, most established with medical schools (13). Intentional distribution of academic research resources throughout all medical education networks would align with World Health Organization (WHO) guidance, rather than retaining expertise in a centralized hub (50). The social accountability framework for medical schools defined by the WHO in 1995 is “the obligation to direct education, *research* and service activities toward *addressing the priority health concerns of the community*, region and/or nation they have a mandate to serve” (51).

## Discussion

Research in place, led from rural locations, by rural researchers engaged with their communities, is critically important. Evidence-based medicine historically excluded women and children, and now we are facing a deficit in evidence on rural populations (1). Distributed health professions education can play a role in closing this gap, toward making evidence-based decisions that are relevant to our full geographic population. Still, solutions must not overlook populations with no trainees, or no university in their jurisdiction. Rurally engaged research is only truly valuable when informed by and conducted by and with communities (17–19). Community engagement to address local priorities may produce more applicable and relevant forms of evidence for health professionals and policy makers (7, 12, 52). Community needs and characteristics will vary, as will the approach to research in each setting and there are no one-size-fits-all solutions. Yet there are overlying principles to support research across rural settings:

1. *Rural questions are legitimate questions and findings are valuable to all.* Due to the interconnected nature of health systems, part of the payoff of improved rural care and outcomes is better coordination with urban providers. There are examples where rural communities are better positioned to innovate and can bring evidence forward to all settings. Frostbite treatment guidelines developed in Whitehorse, and self-collection cervical cancer screening methods validated by First Nations and Metis communities in British Columbia (BC), are just two recent Canadian examples (53–55). The Pentagram Partnership Plus model, developed in BC to address rural and Indigenous health inequities, presents a practical application of social accountability principles with broad applicability (17).
2. *Rural researchers are legitimate researchers and their positionality is of unique value to all research.* They look different from central university-based researchers and the differences are critically important. Until this is acknowledged by scholarly structures and processes, including funding agencies, success will depend too much on individual enthusiasm to grow and to meet the demand for rural evidence. Developing a rural researcher professional identity for rural health professionals in Canada will add to the imperative that rural health research needs rural researchers to do it.
3. *Research infrastructure and processes must reflect the value of rural evidence.* Protected time and support are needed, and the work is rarely done by individuals alone. Distributing the research support team of administrative assistants, research assistants, and methodologists along with education programs, is necessary. Health professions education programs hold many of the needed resources to support the growth of research in these communities and can opt to intentionally distribute more of the resources to rural communities, as has been started in Australia. Robust connectedness of this field will rely on a truly national rural research agenda and network.

From these principles, key priorities emerge: (1) embed rural research into existing systems for long-term sustainability, (2) create avenues for research skills to be taught to rural health professionals and learners by skilled, experienced professionals from similar settings, (3) ensure visibility of rural researchers, (4) support with on-site expertise and navigational knowledge of overall systems, and (5) develop rural research priorities, with funding reflecting the importance of the evidence this work will generate. These actions can shift the positioning of the rural and distributed context to a legitimate academic space where all aspects of academic healthcare occur, and thereby open a rural academic identity to a wide range of rural professionals and increase capacity for their important work.

## Data Availability

The original contributions presented in the study are included in the article/supplementary material, further inquiries can be directed to the corresponding author/s.
